# Feasibility and Acceptability of an Asthma App to Monitor Medication Adherence: Mixed Methods Study

**DOI:** 10.2196/26442

**Published:** 2021-05-25

**Authors:** Cristina Jácome, Rute Almeida, Ana Margarida Pereira, Rita Amaral, Sandra Mendes, Magna Alves-Correia, Carmen Vidal, Sara López Freire, Paula Méndez Brea, Luís Araújo, Mariana Couto, Darío Antolín-Amérigo, Belén de la Hoz Caballer, Alicia Barra Castro, David Gonzalez-De-Olano, Ana Todo Bom, João Azevedo, Paula Leiria Pinto, Nicole Pinto, Ana Castro Neves, Ana Palhinha, Filipa Todo Bom, Alberto Costa, Cláudia Chaves Loureiro, Lilia Maia Santos, Ana Arrobas, Margarida Valério, João Cardoso, Madalena Emiliano, Rita Gerardo, José Carlos Cidrais Rodrigues, Georgeta Oliveira, Joana Carvalho, Ana Mendes, Carlos Lozoya, Natacha Santos, Fernando Menezes, Ricardo Gomes, Rita Câmara, Rodrigo Rodrigues Alves, Ana Sofia Moreira, Diana Bordalo, Carlos Alves, José Alberto Ferreira, Cristina Lopes, Diana Silva, Maria João Vasconcelos, Maria Fernanda Teixeira, Manuel Ferreira-Magalhães, Luís Taborda-Barata, Maria José Cálix, Adelaide Alves, João Almeida Fonseca

**Affiliations:** 1 Center for Health Technology and Services Research Faculty of Medicine University of Porto Porto Portugal; 2 Department of Community Medicine, Faculty of Medicine Information and Health Decision Sciences University of Porto Porto Portugal; 3 Allergy Unit Instituto and Hospital CUF Porto Portugal; 4 Department of Cardiovascular and Respiratory Sciences Porto Health School Porto Portugal; 5 Department of Women’s and Children’s Health Paediatric Research Uppsala University Uppsala Sweden; 6 Servicio de Alergia Complejo Hospitalario Universitario de Santiago Santiago de Compostela Spain; 7 Servicio de Alergia Hospital Universitario Ramón y Cajal Instituto Ramón y Cajal de Investigación Sanitaria Madrid Spain; 8 Serviço de Imunoalergologia Centro Hospitalar e Universitário de Coimbra Coimbra Portugal; 9 Imunoalergologia Centro Hospitalar de Leiria Leiria Portugal; 10 Serviço de Imunoalergologia Hospital de Dona Estefânia Centro Hospitalar Universitário de Lisboa Central Lisboa Portugal; 11 Serviço de Pneumologia Hospital Beatriz Ângelo Loures Portugal; 12 Serviço de Pediatria Hospital da Senhora da Oliveira Guimarães Portugal; 13 Serviço Pneumologia Centro Hospitalar e Universitário de Coimbra Coimbra Portugal; 14 Serviço de Pneumologia Hospital Distrital da Figueira da Foz Figueira da Foz Portugal; 15 Serviço de Pneumologia Hospital Santa Marta Centro Hospitalar Universitário de Lisboa Central Lisboa Portugal; 16 Serviço de Pediatria Hospital Pedro Hispano Unidade Local de Saúde de Matosinhos Matosinhos Portugal; 17 Serviço de Imunoalergologia Hospital de Santa Maria Centro Hospitalar Universitário Lisboa Norte Lisboa Portugal; 18 Serviço de Imunoalergologia Hospital Amato Lusitano Unidade Local de Saúde de Castelo Branco Castelo Branco Portugal; 19 Serviço de Imunoalergologia Centro Hospitalar Universitário do Algarve Portimão Portugal; 20 Serviço de Pneumologia Hospital Garcia de Orta Almada Portugal; 21 Serviço de Imunoalergologia Serviço de Saúde da Região Autónoma da Madeira Funchal Portugal; 22 Serviço de Imunoalergologia Hospital do Divino Espírito Santo Ponta Delgada Portugal; 23 Serviço de Pediatria Unidade Hospitalar de Famalicão Centro Hospitalar do Médio Ave Vila Nova de Famalicão Portugal; 24 Serviço de Pneumologia Hospital Nossa Senhora do Rosário Centro Hospitalar Barreiro Montijo Barreiro Portugal; 25 Serviço de Imunoalergologia Unidade I Centro Hospitalar Vila Nova de Gaia/Espinho Vila Nova de Gaia Portugal; 26 Unidade de Imunoalergologia Hospital Pedro Hispano Unidade Local de Saúde de Matosinhos Matosinhos Portugal; 27 Imunologia Básica e Clínica Faculdade de Medicina Universidade do Porto Porto Portugal; 28 Serviço de Imunoalergologia Centro Hospitalar Universitário de São João Porto Portugal; 29 Serviço de Pediatria Centro Materno Infantil do Norte Centro Hospitalar Universitário do Porto Porto Portugal; 30 Department of Allergy & Clinical Immunology Cova da Beira University Hospital Centre Covilhã Portugal; 31 Environment & Health Study Group, Faculty of Health Sciences Health Sciences Research Centre University of Beira Interior Covilhã Portugal; 32 Serviço de Pediatria Hospital de São Teotónio Centro Hospitalar Tondela–Viseu Viseu Portugal; 33 Serviço de Pneumologia Unidade I Centro Hospitalar Vila Nova de Gaia/Espinho Vila Nova de Gaia Portugal; 34 Medicina, Educação, Investigação, Desenvolvimento e Avaliação Porto Portugal

**Keywords:** mHealth, smartphone, technology assessment, medication adherence, self-management, gamification, patient participation

## Abstract

**Background:**

Poor medication adherence is a major challenge in asthma, and objective assessment of inhaler adherence is needed. The InspirerMundi app aims to monitor adherence while providing a positive experience through gamification and social support.

**Objective:**

This study aimed to evaluate the feasibility and acceptability of the InspirerMundi app to monitor medication adherence in adolescents and adults with persistent asthma (treated with daily inhaled medication).

**Methods:**

A 1-month mixed method multicenter observational study was conducted in 26 secondary care centers from Portugal and Spain. During an initial face-to-face visit, physicians reported patients’ asthma therapeutic plan in a structured questionnaire. During the visits, patients were invited to use the app daily to register their asthma medication intakes. A scheduled intake was considered taken when patients registered the intake (inhaler, blister, or other drug formulation) by using the image-based medication detection tool. At 1 month, patients were interviewed by phone, and app satisfaction was assessed on a 1 (low) to 5 (high) scale. Patients were also asked to point out the most and least preferred app features and make suggestions for future app improvements.

**Results:**

A total of 107 patients (median 27 [P25-P75 14-40] years) were invited, 92.5% (99/107) installed the app, and 73.8% (79/107) completed the 1-month interview. Patients interacted with the app a median of 9 (P25-P75 1-24) days. At least one medication was registered in the app by 78% (77/99) of patients. A total of 53% (52/99) of participants registered all prescribed inhalers, and 34% (34/99) registered the complete asthma therapeutic plan. Median medication adherence was 75% (P25-P75 25%-90%) for inhalers and 82% (P25-P75 50%-94%) for other drug formulations. Patients were globally satisfied with the app, with 75% (59/79) scoring ≥4,; adherence monitoring, symptom monitoring, and gamification features being the most highly scored components; and the medication detection tool among the lowest scored. A total of 53% (42/79) of the patients stated that the app had motivated them to improve adherence to inhaled medication and 77% (61/79) would recommend the app to other patients. Patient feedback was reflected in 4 major themes: medication-related features (67/79, 85%), gamification and social network (33/79, 42%), symptom monitoring and physician communication (21/79, 27%), and other aspects (16/79, 20%).

**Conclusions:**

The InspirerMundi app was feasible and acceptable to monitor medication adherence in patients with asthma. Based on patient feedback and to increase the registering of medications, the therapeutic plan registration and medication detection tool were redesigned. Our results highlight the importance of patient participation to produce a patient-centered and engaging mHealth asthma app.

## Background

Inhaled preventive medications are the cornerstone of effective asthma treatment, reducing symptoms and exacerbations [1]. Yet in the last three decades, rates of adherence among patients with asthma remained unchanged, ranging from 19% to 64% [2]. Poor medication adherence is thus a major challenge in asthma [2,3] and is a leading driver of poor health outcomes, including emergency department visits, hospitalizations, and increased health care costs [4]. Based on this significant burden, medication adherence is a top priority for disease management [1] and policy agendas [5,6].

Research efforts to tackle medication nonadherence have not led to the much needed improvement [7]. Behavioral and educational interventions had modest effects, possibly because most propose one-size-fits-all solutions for distinct nonadherence phenotypes (erratic, unwitting, intelligent) [8]. These interventions also failed to consider facilitators for real-world implementation [8]. The objective measurement of inhaler adherence is another major problem [9]. Electronic monitoring devices are available but their dissemination in real-world practice is difficult and costly [10].

Mobile health (mHealth) technologies may be promising to support medication adherence as they are easily integrated into patients’ everyday lives, especially if using only smartphone embedded sensors [11]. mHealth allows the combination of computation, communication, and interactive display that makes it possible to use gamification and peer support tools with the potential to inspire behavior changes and offer treatment adherence quantification [11,12]. Moreover, patients with asthma have increasingly high smartphone ownership levels and are interested in using disease-specific apps [13,14].

The InspirerMundi app development was grounded in previous research and cooperation with patients with asthma and physicians [15]. The app aims to transform adherence to inhalers into a positive experience through gamification and social support while allowing for verified inhaler adherence monitoring. The concept and gameplay of the InspirerMundi app derive from promoting user social interaction in a quest to help other users increase inhaler adherence (while being an example of good adherence). The user’s goal is to become an Inspirer to increase the sphere of positive influence by coaching an expanding network of Warriors (as Warriors progress and keep good medication adherence, they can become Inspirers themselves). The most innovative app technology is the medication detection tool based on advanced processing of inhaler images captured with the smartphone camera [16].

The app development process was iterative with real-life tests and improvements based on user feedback. The first version was previously tested in a usability study, and improvements were made [17]. It is believed that the InspirerMundi app is innovative in measuring and improving medication adherence in patients with asthma. Before studying its effectiveness, however, there is a need for feasibility studies to monitor its use and evaluate its acceptability. Thus, this study aimed to evaluate the feasibility and acceptability of the InspirerMundi app to monitor medication adherence in adolescents and adults with asthma.

## Methods

### Study Design

A multicenter observational study with one initial face-to-face visit and a telephone interview at 1 month was conducted [18]. During the study, an additional telephone interview at 1 week for additional data collection and 3 text messages (at 2, 14, and 21 days) to promote app engagement were added. A convenience sample of adolescents and adults with persistent asthma was recruited between November 2017 and April 2019 at 26 allergy, pulmonology, and pediatric secondary care centers in Portugal (North, Centre, Lisbon, Algarve, Azores, and Madeira regions) and Spain (Galicia and Madrid regions). Centers were asked to recruit at least 2 patients as a minimum of 20 adolescents and 20 adults were targeted [19,20]. The study protocol was approved by the ethics committees of all participating centers. The study was conducted in accordance with the ethical standards established in the Declaration of Helsinki. Eligible patients were approached by physicians during medical visits and invited to participate in a study on the use of an app to register their asthma medication daily intake. Written informed consent was obtained before enrollment in the study. Adult patients signed a consent form; adolescents signed an assent form and a parental consent form was also obtained. The study is reported according to STROBE (Strengthening the Reporting of Observational Studies in Epidemiology) guidelines [21].

### Participants

Patients were included if they (1) had a previous medical diagnosis of persistent asthma, (2) were at least 13 years old (13 to 17 years for adolescents; 18 years and older for adults), (3) were able to use mobile apps and had access to a mobile device with internet access, and (4) had an active prescription for a daily inhaled controller medication for asthma. All inhaled controller treatments were allowed, and there was no change in any prescribed medication as a result of the participation in this study. Patients were excluded if they had a diagnosis of a chronic lung disease other than asthma or a diagnosis of another significant chronic condition with possible interference with the study aims.

### InspirerMundi App

The InspirerMundi app is focused on supporting patient medication management and promoting treatment adherence. The InspirerMundi app is grounded on the Fogg behavior model, which states that a behavior is performed when 3 elements converge in a given moment: motivation, ability, and trigger (Table 1) [22]. The app includes registration of the therapeutic plan, which activates notifications when a medication is due (trigger) and allows recording of performed intakes through a medication detection tool that uses the smartphone camera. The medication detection tool is based on advanced image processing techniques and identifies the inhaler through template matching [15]. At the time of the study, the tool could detect 6 inhaler devices containing numeric dose counters (Diskus/Accuhaler [GlaxoSmithKline PLC], Ellipta [GlaxoSmithKline PLC], Flutiform pMDI [Napp Pharmaceuticals LTD], Novolizer/Genuair [AstraZeneca], Spiromax [Teva Pharmaceutical Industries LTD], Turbohaler [AstraZeneca]). The medication detection tool can also be applied to other types of inhalers or other drug formulations (eg, blister or other recipients) but the template matching feature cannot. In the app version tested, a scheduled intake was considered taken when patient presented the medication (inhaler, blister, or other drug formulation) to the detection tool (10 seconds). Adherence statistics are provided to the user in the form of circular progress graphs (motivator: self-monitoring; ability: simplifies adherence measurement). Besides planned medication, the user may also record relief medication intake events that are not considered for treatment adherence assessment. Physician involvement is also supported by providing the user with the possibility of sharing the registered therapeutic plan and respective adherence data. Medication management is supported by a timeline that reflects the registered therapeutic plan. The timeline also includes events related to gamification and symptom monitoring. Patients are invited to answer 3 types of questionnaires related to their symptoms and asthma control: daily questionnaire (daily after 6 PM), weekly questionnaire (once per week), and Control of Allergic Rhinitis and Asthma Test (CARAT, by default once per month but can be personalized by the patient to weekly or every 2 weeks) [23] (motivator: self-monitoring, personalization; ability: simplifies assessment of asthma control). CARAT total score is calculated by summing the 10 questions resulting in a range of 0 to 30 points. A score >24 indicates good disease control [24], and this classification is presented to the patient (motivator: anticipation/hope of a health improvement). This questionnaire is available in multiple languages, and its psychometric properties are established [25].

**Figure 1:**
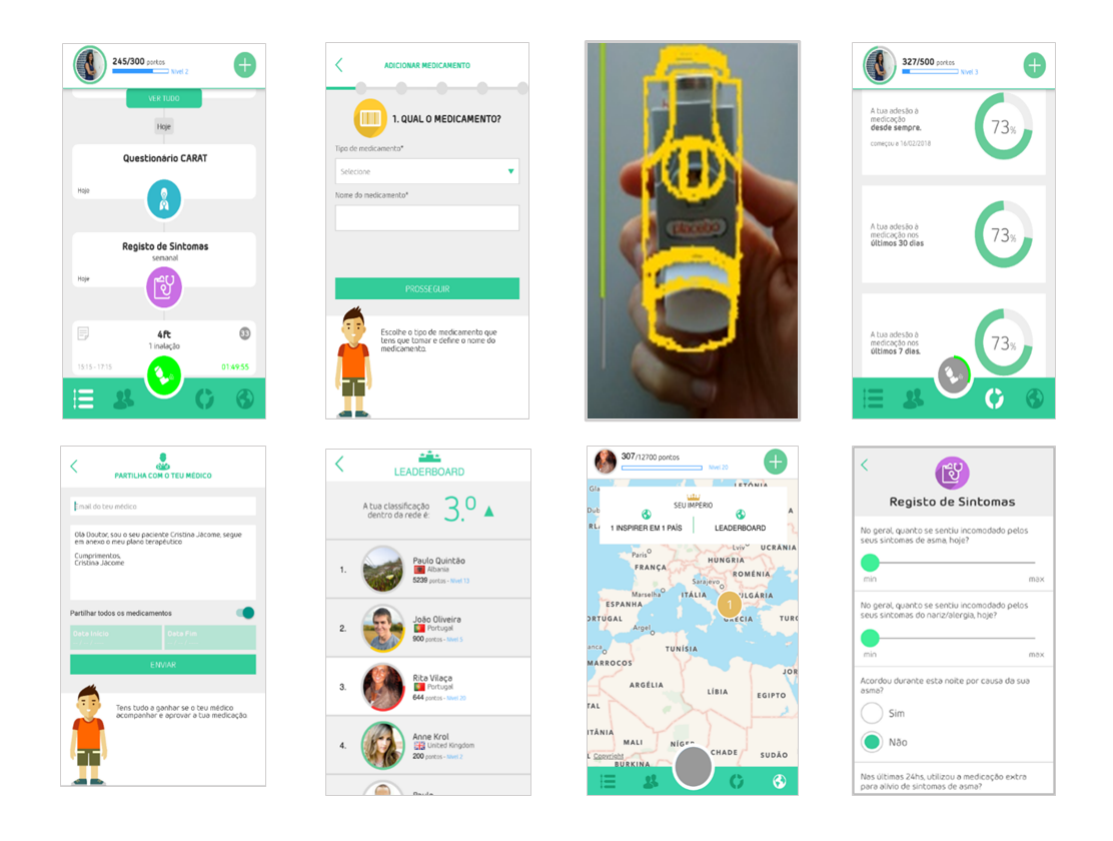
InspirerMundi app screenshots (version 1.1).

The concept and gamification dynamics are twofold: achievement challenges and social interaction (motivator: belonging, social acceptance, app as a social actor). Gameplay and mechanics included in the app derive from achievement points and badges (triggers) and by promoting social interaction based on a story of evolving from a Warrior (beginner player, level 1) to an Inspirer (advanced player, an example of good adherence, available from level 10 onward), who can motivate Warriors to improve adherence (motivators: cooperation, competition, recognition). The main components that support the game are points earned when users take certain actions (motivator: sensation of pleasure, positive reward) such as registering a new medication, registering scheduled medication intakes at the right time, answering symptom questionnaires, or getting a positive assessment from other users in their network. Registering events of relief medication does not generate additional points. When registering a relief medication intake, patients are invited to rate the severity of symptoms experienced on a visual analog scale (VAS; 0 to 100) and report any use of health care. Another encouragement for user interaction is the attribution of virtual badges (12 available; motivator: sensation of pleasure, positive reward). Whenever the user reaches a certain goal (eg, Role Model badge when the user links to the first Warrior) or does something special (eg, Big Influencer badge when the user reaches 5 Warriors), they will be rewarded and get a virtual badge for their actions. The app, developed in Portuguese, English, and Spanish, is available in the App Store (version 1.1) and Google Play (versions 1.1.x); app is only available for download in Portugal and Spain. Screenshots of the app version tested (version 1.1) are presented in Figure 1, and videos are available [26-29].

**Table 1:** Elements of the Fogg behavior model in the InspirerMundi app.

Elements and description of the Fogg behavior model			App feature		App component
**Trigger**					
	Facilitator of adherence	Signal: notification appears when a medication intake is due		Medication management	
	Facilitator of medication intake registration	Signal: intake icon flashes when a medication intake is due		Medication management	
	Facilitator of asthma control assessment	Signal: questionnaire icon flashes when a response is due		CARAT^a^, daily questionnaire, and weekly questionnaire	
	Facilitator of adherence	Signal: buzzes when an Inspirer sends a medication reminder to a Warrior		Medication management, gamification	
	Facilitator of adherence	Signal: icon of message appears (exchange between Inspirer and Warrior)		Gamification	
**Ability**					
	Simplifies adherence measurement	Adherence statistics: circular progress graph		Medication management	
	Simplifies assessment of asthma control	Asthma control classification		CARAT	
**Motivator**					
	Self-monitoring	Adherence statistics: circular progress graph		Medication management	
	Self-monitoring, personalization	Personalized frequency		CARAT	
	Anticipation/hope of a health improvement	Asthma control classification		CARAT	
	Belonging, social acceptance, app as a social actor	Social interaction		Gamification	
	Cooperation, competition, recognition	Inspirer and Warrior players		Gamification	
	Sensation of pleasure, positive reward	Points		Gamification	
	Sensation of pleasure, positive reward	Virtual badges		Gamification	

^a^CARAT: Control of Allergic Rhinitis and Asthma Test.

### Data Collection

During the initial face-to-face visit, physician recorded if it was a first or follow-up appointment; patient’s asthma control according to the Global Initiative for Asthma (GINA) [1]; number of exacerbations in the previous year (defined as episodes of progressive increase in shortness of breath, cough, wheezing, and/or chest tightness requiring a change in maintenance therapy [30]); and use of health care resources in the previous year (ie, number of unscheduled medical visits to primary care, secondary care, or emergency department and number of hospital admissions). Physician also recorded patient’s current asthma treatment, including inhaled and oral medication, allergen immunotherapy, and biologic therapy. At the visit, patient answered written questionnaires on demographic data (age, gender, BMI, smoking habits), adherence to inhaled controller asthma medication during the previous week, and perception of the correctness of inhaler technique (using a 100 mm VAS), asthma control during the previous 4 weeks (using CARAT), perception of their overall health (using the EuroQol 5-Dimension [EQ-5D] VAS [31]), and previous use of health and fitness apps.

At 1 week and 1 month, patients were asked in a telephone interview about their adherence to inhaled controller medication during the previous week (on a 0 to 100 scale) and their asthma control with CARAT. At 1 month, they were asked to rate the app usability using 4 items of the System Usability Scale (ease of use, function integration, confidence in using, would use frequently) [32]. Patients were also asked about their general satisfaction with the app and app components (gamification, peer support, symptom monitoring, medication detection tool, adherence monitoring), their willingness to recommend the app to others, and the impact of the app on the motivation to adhere to the inhaler. All these satisfaction questions were assessed using a 1 (low) to 5 (high) scale. Patients were asked open questions on the most and least preferred app features and asked to make suggestions for app improvements. These telephone interviews could also provide support regarding any technical issues with the app or study-related doubts. Nonscheduled telephone contacts could also occur, as patients were provided with email and telephone contacts to use in case of any app technical issue; this was rarely used. Interviews were performed by a central team of trained health professionals and lasted around 15 minutes. An interview guide was used to standardize data collection across all patients.

### Data Analysis

Descriptive statistics were used to characterize the sample and app use. The normality of each variable was investigated with Kolmogorov-Smirnov tests and visual analysis of histograms. The app use rate was taken as the ratio between the number of days with app use and the period of use (31-day period). Adherence to medication measured by the app was calculated using 2 methods: considering the medication taken only on days with app use and considering the medication taken regardless of app use (ie, considering all medication scheduled for the 31-day period). Comparison of the characteristics of patients installing the app versus those not installing, using the app for more than 1 day versus single-day use, completing the study versus dropouts, and adolescents versus adults were performed using independent samples *t* tests, Mann-Whitney *U* tests for continuous variables, and chi-square tests for categorical variables. A Fisher test was used to compare adherence to inhalers measured by the app with patient self-reports (at the initial visit, 1 week, and 1 month). The Wilcoxon test and McNemar test were used to analyze differences in CARAT total score and CARAT control classification, respectively. Statistical analyses were performed using SPSS (version 26.0, IBM Corp) and plots were created using GraphPad Prism (version 6.0, GraphPad Software). The level of significance was set at .05. A thematic qualitative analysis of patient feedback (ie, opinions, suggestions) about the app collected during the 1-month telephone interview was also conducted. Notes from the interviews were read, and emerging themes were grouped. Description of patient feedback in major themes is supported with translated representative statements.

## Results

### Participants

A total of 107 patients were included: 77 from 24 Portuguese centers and 30 from 2 Spanish centers. Patients included mostly adults (77/107; 72.0%) and females (63/107; 58.9%). Most were on inhaled corticosteroid/long-acting beta-agonist combination therapy (90/107; 84.1%) and used only one inhaler (67/107; 62.6%). Based on GINA classification, almost half (52/107; 48.6%) of the participants did not have their asthma well controlled. Characteristics of the participants are shown in Table 2.

**Table 2:** Participant baseline characteristics (n=107).

Characteristic			Total (n=107)		Did not install app (n=8)		App used 1 day (n=25)	App used >1 day (n=74)
Age (years), median (P25-P75^a^)			27 (17-40)		40 (28-46)		22 (16-40)	26.5 (17-39)
Adults, n (%)			77 (72.0)		8 (100)		15 (60)	54 (73)
Female, n (%)			63 (58.9)		6 (75)		10 (40)	47 (64)
BMI (kg/m^2^), median (P25-P75)			23.1 (21-27)		26.2 (22-27)		24 (22-29)	22.8 (21-26)
**Smoking status, n (%)**								
	Never smoker	79 (73.8)		5 (63)		17 (68)		57 (77)
	Ex-smoker	19 (17.8)		3 (37)		5 (20)		11 (15)
	Current smoker	9 (8.4)		0		3 (12)		6 (8)
**Inhaled medication, n (%)**								
	ICS^b^/LABA^c^	90 (84.1)		6 (75)		23 (92)		61 (82)
	SABA^d^	26 (24.3)		3 (38)		9 (36)		14 (19)
	ICS	13 (12.1)		1 (13)		2 (8)		10 (14)
	LAMA^e^	10 (9.3)		0		1 (4)		9 (12)
Single inhaler, n (%)			67 (62.6)		4 (50)		15 (60)	48 (65)
**VAS^f^ self-reported inhaler adherence (0-100)^g^**			80 (60-90)		83 (59-91)		79 (53-90)	85 (65-90)
	High (81-100), n (%)	51 (47.7)		4 (50)		7 (28)		40 (54)
	Medium (51-80), n (%)	37 (34.6)		3 (38)		12 (48)		22 (30)
	Low (0-50), n (%)	15 (14.0)		1 (12)		4 (16)		10 (14)
VAS correctness of inhaler technique (0-100)			99 (90-100)		92.5 (89-99)		90 (80-100)	99.5 (90-100)
Oral medication, antileukotriene, n (%)			42 (39.3)		0		8 (32)	34 (46)
Allergen immunotherapy, n (%)			19 (17.8)		2 (25)		5 (20)	12 (16)
Biologic therapy, n (%)			8 (7.5)		0		1 (4)	7 (9)
**GINA^h^ assessment symptom control^i^, n (%)**								
	Well controlled	53 (49.5)		3 (38)		11 (44)		39 (53)
	Partly controlled/uncontrolled	52 (48.6)		4 (50)		13 (52)		35 (47)
CARAT^j^ total score, mean (SD)			21 (17-23)		14.5 (9-27)		20 (16-22)	21 (17-24)
≥1 exacerbation past year, n (%)			52 (48.6)		6 (75)		16 (64)	30 (41)
≥1 unscheduled medical visit past year, n (%)			27 (25.2)		2 (25)		10 (40)	15 (20)
EQ-5D^k^ VAS, mean (SD)			80 (70-90)		80 (58-89)		75 (65-85)	82 (70-90)
First medical visit, n (%)			23 (21.5)		4 (50)		8 (32)	11 (15)
Previous use of health and fitness apps, n (%)			58 (54.2)		5 (63)		12 (50)	41 (55)

^a^P25-P75: percentile 25 to percentile 75.

^b^ICS: inhaled corticosteroid.

^c^LABA: long-acting beta-agonist.

^d^SABA: short-acting beta-agonist.

^e^LAMA: long-acting muscarinic receptor antagonist.

^f^VAS: visual analog scale.

^g^4 missing values.

^h^GINA: Global Initiative for Asthma.

^i^2 missing values.

^j^CARAT-T: Control of Allergic Rhinitis and Asthma Test total score.

^k^EQ-5D: EuroQol 5-Dimension test.

### App Use

**Figure 2:**
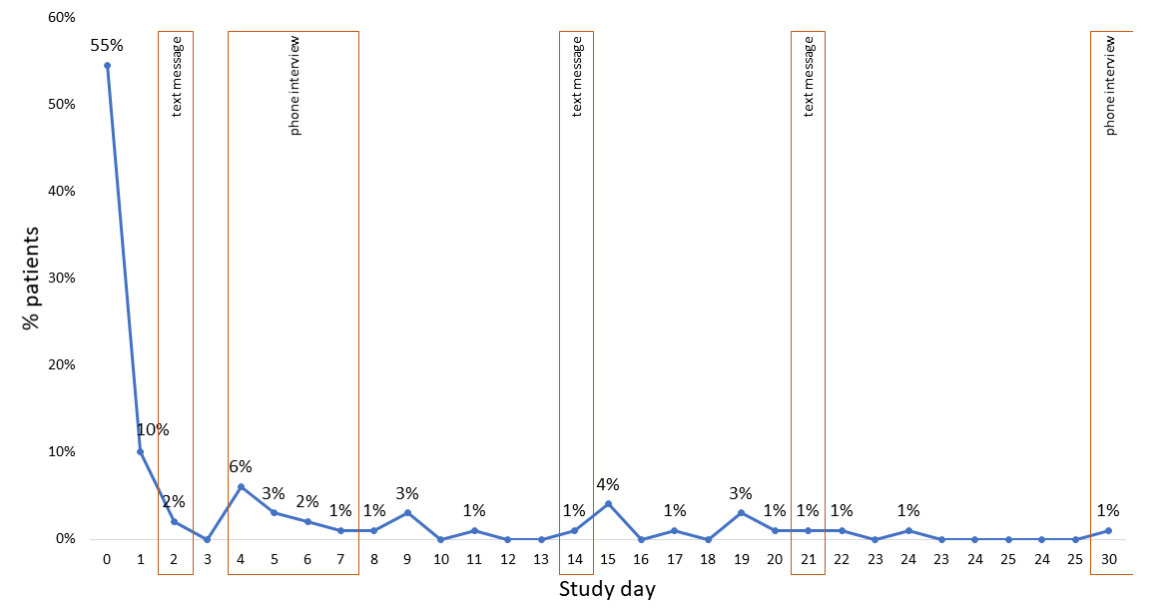
Percentage of users installing the app per study day.

In total, 92.5% (99/107) of patients installed the app. The 8 patients who did not install the app were more frequently in a first medical appointment with that physician (*P*=.02) and tended to be older (*P*=.05) than those who installed the app. No other significant differences were observed between the 2 groups. More than half (64/99; 65%) of patients installed the app on the recruitment day or the following day (median 0 [P25-P75 0-5]; Figure 2).

**Figure 3:**
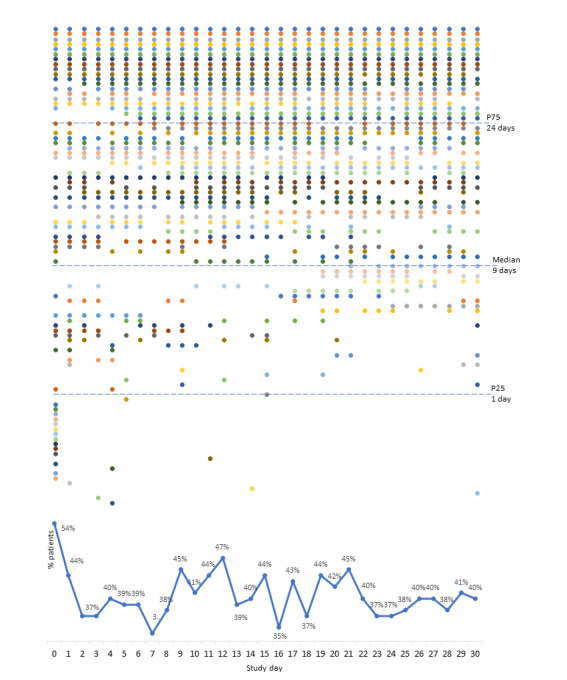
Daily participant engagement with the InspirerMundi app (n=97, 2 users with missing information). Each dot represents a day with interaction, and each color represents a participant.

Patients interacted with the app a median of 9 (P25-P75 1-24) days: 25% (25/99) only 1 day and 10% (10/99) all 31 days (Figure 3). A median use rate of 29% (P25-P75 3%-77%) was observed. Patients interacting only 1 day were more frequently male (*P*=.04), had a worse perception of their health status (*P*=.045), and had more exacerbations in the previous year (*P*=.04) in comparison with patients interacting more than 1 day. Although not reaching statistical significance, these patients had a higher need for unscheduled medical visits in the previous year (*P*=.05), more frequent short-acting beta-agonist prescribed in their therapeutic plan (*P*=.09, physician-reported), and more frequent depression symptoms (*P*=.09).

A total of 78% (77/99) of patients scheduled medication in the app (median 2 [P25-P75 1-3] medications) for 25 (P25-P75 17-31) days: 74% (73/99) registered at least 1 inhaler and 42% (42/99) at least another medication (eg, pills, nasal spray). The asthma therapeutic plan reported by the physician included a median of 2 (P25-P75 1.8-2) medications. A total of 53% (52/99) of participants registered all prescribed inhalers and 34% (34/99) registered the complete asthma therapeutic plan (inhaler and other drug formulations). Two (2%) patients also used the app to register medications not related to asthma.

Median inhaler adherence assessed through the app was 81% (P25-P75 58%-92%) when considering only days with app use and 62% (P25-P75 23%-81%) when considering all scheduled inhalations for the 31-day period (*P*<.001). Higher estimates were observed when patients self-reported adherence at initial visit (85 [P25-P75 63-90]), 1-week (95 [P25-P75 80-100]) and 1-month (100 [P25-P75 90-100]) interviews (*P*<.001; all pairwise comparisons *P*<.009 with exception of 1-week and 1-month interview that were not different). Median adherence for other drug formulations was 83% (P25-P75 50%-94%) when considering only days with app use and 73% (P25-P75 26%-94%) when considering all scheduled inhalations for the 31-day period (*P*<.001).

A total of 17% (17/99) registered the use of relief medication for at least 1 day (median 1 [P25-P75 1-2]): 13% (13/99) used inhalers, 4% (4/99) other drug formulations (pills, nasal spray), and 1% (1/99) inhaler plus others. During these episodes, symptom severity reached a median VAS of 43 (P25-P75 29-60), with 2% (2/99) of patients reporting emergency department visits and 1% (1/99) an unscheduled medical appointment.

A total of 63% (62/99) of patients answered symptom questionnaires: 62% (61/99) answered at least one CARAT (median 1 [P25-P75 1-2]), 58% (57/99) answered weekly symptom questionnaires (median 4 [P25-P75 2-4]), and 42% (42/99) answered daily symptom questionnaires (median 9.5 [P25-P75 4-13.3]). A total of 76% (75/99) received at least one badge (2 [P25-P75 2-3]), 63% (62/99) achieved 1000 points, and 21% (21/99) achieved 10,000 points. A total of 6% (6/99) of users became Inspirers, a role achieved between 5 and 28 days of app use.

### App Satisfaction

**Figure 4:**
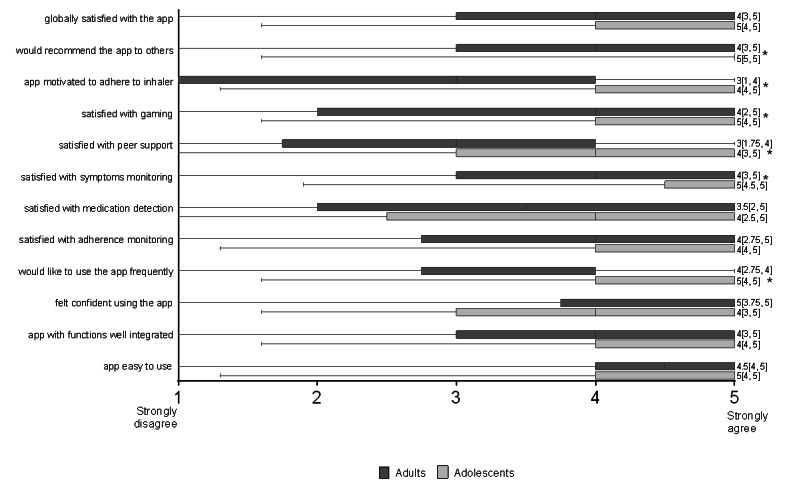
App feedback from adolescents and adults (n=79). Data are presented as box plots (lines inside the boxes represent the medians; bounds of boxes, first and third quartiles; bars, 95% confidence interval). *Significant differences between adolescents and adults.

A total of 74% (79/107) of patients completed the 1-month interview (28/107; 26% dropout rate). After 1 month, CARAT scores improved (21 [P25-P75 16-23] vs 25 [P25-P75 22-27]; *P*<.001), with the proportion of patients with controlled asthma increasing from 20% (16/79) to 53% (42/79; *P*<.001). Patients were globally satisfied with the app, with 75% (59/79) scoring ≥4 on a 1 to 5 scale (33% [26/79] scored 4 and 42% [33/79] scored 5), with the adherence monitoring, symptom monitoring, and gamification being the most highly scored components (Figure 4). Only 14% (11/79) were not satisfied with the app (score ≤2). Patients found the app easy to use, considered the functions well integrated, felt confident using the app, and would like to use it frequently. A total of 77% (61/79) of patients would recommend the app to other patients, and 53% (42/79) believed the app had motivated them to improve adherence to inhaled medication. In general, adolescents were more satisfied with the app than adults (Figure 4).

### App Features Feedback

All patients provided feedback on the app, 95% (75/79) pointed out the aspects that they valued most, 73% (58/79) mentioned aspects least preferred, and 62% (49/79) made improvement suggestions. Patient feedback was reflected in 4 major themes: medication-related features (67/79, 85%), gamification and social network (33/79, 42%), symptom monitoring and physician communication (21/79, 27%), and other aspects (16/79, 20%). These themes are described in detail below.

#### Medication-Related Features

Patients pointed out the notifications for the medication (47/79, 59%) and adherence statistics (18/79, 23%) as strengths of the app. Yet 2 patients reported problems in receiving notifications, and 2 suggested improvements such as making notifications clearer, sending a second notification if the medication was not taken within 1 hour, and allowing the confirmation of medication intake without entering the app. The available time to confirm the medication intake (2 hours after the notification) was considered inflexible (13/79, 16%) as it did not allow for confirmation if the medication was taken earlier (before the notification) or later. This was seen as a disadvantage as it compromised the game and adherence statistics. Patients asked for the possibility to also confirm medications when outside of scheduled hours (15/79, 19%).

[Notifications] It helped me to remember to take the medication.

With the adherence statistics, I have a better perception of my real adherence.

I lost points because I took the medication out of the scheduled hours, and I was not able to confirm it.

The need to use the medication detection tool to confirm each medication intake was seen as a weak point (22/79, 28%), mainly due to the predefined time to capture the image, perceived as long. This was especially burdensome for patients with more complex medication regimens (eg, more than one control medication per day). Patients suggested that the medication detection tool could be faster in detecting the dose counter (5/79, 6%), provide feedback of the identified number (2/79, 3%), and be activated only at certain time points (eg, every 5 days or with personalized frequency; 4/79, 5%). Two patients would like the app to include educational content related to asthma and inhalation technique.

The time I need to wait for the app to take the picture of the inhaler...

There could be an option that allows you not to have to take a photo of the inhaler daily, but to take it, for example, on the first day and after 5 or 7 days.

The app could include a demonstration on how to use the inhaler using images.

Registration of controller or relief medication was reported as difficult by 4 patients and they asked for a simpler process, suggesting, for example, selecting the medication from a provided list, not needing to insert a new medication when a new inhaler is bought, and personalizing scheduled times depending on the day of the week (eg, during weekends).

I found it difficult to register an SOS intake; I need more information to be able to use this feature.

When the inhaler is over, it would be nice not to have to register a new inhaler with all the data.

#### Gamification and Social Network

Opinions differed with regard to the gamification and social network components: 18% (14/79) of patients (both adolescents and adults) were happy and stated that these components were an additional motivation to improve adherence; 13% (10/79, 9 adults) felt these components were unnecessary or not adding benefit. Two patients reported the gamification approach as confusing, and others did not like the Inspirers avatar.

The game increases my motivation to take the medication.

I don’t have any interest in using the game or to share experiences in the social network.

#### Symptom Monitoring and Physician Communication

Patients pointed out the advantages of symptom monitoring (10/79, 13%), with three acknowledging that it increased their perception of disease control (n=3). Three patients also highlighted the utility of the app to share clinical information (use of relief medication, symptoms) with the physician (n=3). One patient suggested a more direct connection with the physician through a chat, for example, to receive recommendations, clarify doubts, and even schedule appointments, and another suggested the addition of features to compare symptom evolution.

I liked the symptom questionnaire and interpretation. The questionnaires were simple to fill in.

The possibility to have a reliable registration of my disease trajectory and to be able to show to a health professional. I hope to have benefits with this registration!

It would be good to chat with my physician, clarify doubts, and schedule appointments.

I would like to compare the results of the questionnaires to be able to notice if there was any improvement in symptoms or not.

#### Others

Patients reported experiencing technical difficulties/bugs (during log-in, registration of symptom questionnaires, medication detection tool; sometimes the app crashed or closed unexpectedly [8/79, 10%]) and two patients reported that the app used significant storage space (n=2). Other suggestions were a timeline showing the present events by default, simplified log-in (eg, touch ID), and improved access to Definitions and Profile menus.

The app could be smaller.

Log-in could be simplified, and could be with smartphone biometry.

### App Changes Based on Patient Use and Feedback

Based on patient feedback, two major changes were implemented in a new Android app (version 1.2): redesign of the therapeutic plan registration module and medication detection tool. Now, to register an inhaler, the patient is provided with images of the most common inhalers available on the Portuguese and Spanish markets and once the patient selects the inhaler type, a drop-up list presents all names/dosages of medications available on the market. The patient can also select different schedules on different days, allowing the personalization requested. To allow the registration of the complete asthma therapeutic plan, the patient can indicate if the controller inhaler can also be used as relief and how many inhalations are recommended in that case. Also, as registering medications is the only way the app produces notifications, this is now the first task asked once the patient enters the app.

**Figure 5:**
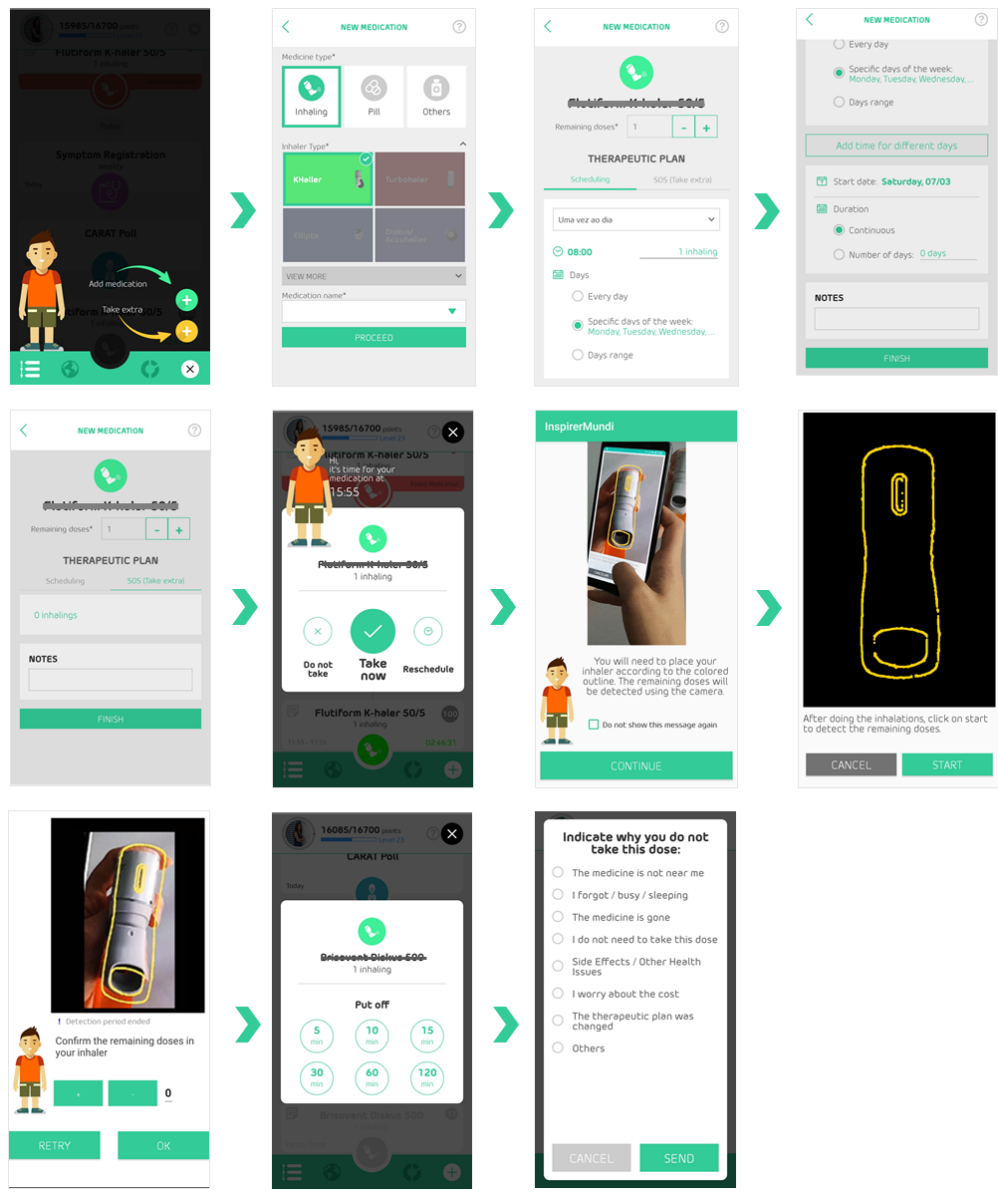
InspirerMundi app screenshots (version 1.2).

When a medication is due, notifications provide information on the medication and posology and the patient can select 1 of 3 options: confirm medication was taken, delay medication intake (from 5 to 120 minutes), or decide not to take it. Patients now have until the end of the day to confirm medication was taken. The medication detection tool is currently able to detect 12 inhaler devices containing numeric dose counters available on the Portuguese and the Spanish markets. The medication detection tool is activated daily only for inhalers (not for other drug formulations) to reduce the users’ burden. When the patient confirms medication was taken, an initial screen with instructions on how to match the inhaler with the template is provided, but in the current version instead of an example figure, we use a short video demonstrating the task (patients can decide to inactivate these instructions whenever they want to accelerate the process). A feature to provide feedback on the identified number in the inhaler was already developed [16] allowing the user to correct the number, and it will be introduced in a future app version. Reasons for deciding not to take a medication can also be registered (eg, medication is empty, this dose is not needed, secondary effects). Screenshots with these app changes are presented in Figure 5, and videos are available [33,34].

## Discussion

### Principal Findings

The InspirerMundi app was found to be feasible to monitor medication adherence and positively evaluated by patients with persistent asthma. The results of this study provide an understanding of the feasibility and acceptability of this mHealth technology in this population. The study contributed to identify improvements for a better user experience before effectiveness studies are undertaken.

Patient use rate of the app is similar to a previous mHealth asthma observational study (median of 12 days in the first 3 months) [35,36], and it is within the range described in digital health studies (median 6 days, range 2 to 26, in the first 3 months) [36]. Nevertheless, we consider the patient use rate lower than expected as the app was directly recommended by a physician and app use was reinforced by 3 text messages and a telephone interview. This low engagement may be linked to the real-world nature of these studies and the low previous use of asthma apps (<5%) [13]. Importantly, it may also be related to the low level of maturity of some of the app technologies. Less than one-third of patients interacted with the app only 1 day, which is lower than previously described behaviors [36]. These patients were found to be the ones with worst asthma outcomes, such as unscheduled medical visits and exacerbations, and worst perception of health status. Chan et al [35] also observed that the group of patients with fewer interactions with an asthma app were the ones with higher hospitalization rates and emergency department visits. Male patients were also associated with lower interaction, in accordance with previous observations of male patients to be less likely to adopt health apps [37]. These observations need to be considered in the design of mHealth-based interventions to improve adherence to medications.

A small proportion of patients entered the study but did not install the app (8%), which is in line with previous studies with asthma apps [38]. It was not surprising to observe that these patients were older and most commonly were in a first medical appointment. In fact, older patients are the least represented in digital health studies [36,39] and are also less likely to adopt health apps [37]. First appointments with a health care provider are linked to a greater discordance between patients and physicians [40], which may contribute to reducing the known role of physician recommendation as a facilitator of mHealth adoption [36,41]. The total dropout rate (26%) is in accordance with rates in feasibility studies with digital interventions [42] but higher than previously described in clinical trials [38]. This dropout rate was expected given the real-world nature of the feasibility study involving patients from a large number of secondary care centers (26 in total), very diverse in terms of settings—secondary and tertiary care, public and privately owned, dimension, geographic regions, and medical specialties (allergy, pulmonology, and pediatrics). This is contrasted with clinical trials, which are most often conducted in well-selected academic centers with more homogeneous samples and a high number of face-to-face visits.

More than three-quarters of patients used the app for medication management and adherence monitoring, which were the primary aims of app use, and a median score of 4 out of 5 was obtained for this app feature. Self-reported inhaler adherence at the initial visit, 1-week and 1-month interview overestimated adherence measured with the app. This was somewhat expected as self-reports had a near to perfect treatment adherence behavior, which is unlikely to be the case [40]. Overestimation of adherence measured by the app is also possible, as no objective confirmation of the inhaler dose counter was performed in this version. New versions of the app should assess inhaler adherence more objectively by validating inhaler use through dose tracking. The medication detection tool is currently working with 13 inhaler devices containing numeric dose counters available on the Portuguese and Spanish markets [16]. To improve the generalizability of the app and its use in multicenter international studies, inhalers available in other countries will be added in future versions of the app.

Patient evaluations were positive: three-quarters were globally satisfied (75%), reporting scores of 4 (33%) and 5 (42%). The InspirerMundi app acceptability was similar to that described in other feasibility studies of mHealth apps [43-45]. Notifications for medication intake were considered helpful [20,46]. Patients made important contributions for improvement such as the need to make notifications clearer and registration of medications easier. These requested changes are in line with user reviews for other adherence apps [46] and were implemented in the InspirerMundi Android version 1.2, currently being tested by patients recruited at primary care centers. Patients also suggested improvements to the medication detection tool, which led to the tool’s redesign, including feedback on the identified number.

Symptom monitoring, used by almost two-thirds of patients, was one of the most highly scored components, with patients stating that it increased their perception of asthma control [20]. Patients valued the feature of sharing information with physician [20,46] and requested other patient-physician partnership tools. In the tested version, patients could send data on the therapeutic plan registered in the app and respective adherence to their physician by email. New simple and easily read reports (including graphs/charts) have been designed, and a tool for remote personalized feedback is being planned. We believe these upgrades will facilitate the integration of app data into clinical workflow and strengthen the patient-physician partnership.

Based on patient feedback, improvements on gamification, social support and monitoring features, and integration of educational content are needed to ensure ongoing digital engagement. The team is considering the integration of new gamification dynamics and mechanics (eg, avatar personalization, progression bars, boosters, exchange points, other tangible rewards), social support features (eg, discussion forums, mentoring, group actions), and educational features (eg, training videos on inhalation technique) [20]. There were opposing opinions regarding the gamification and social network components, also observed in a previous study with adolescents with asthma [20]. To accommodate these differences, and as personalized strategies to treatment adherence have better results [47,48], gamification and social network features of the app will become optional and patients not willing to use these features will be able to turn them off. This modification is in line with previous work advocating that flexible apps may better meet the needs of a broader range of patients [49,50].

### Limitations

This study has limitations. As a feasibility study, a control group was not included, and the sample size was not powered. This was a deliberate choice to test the feasibility and acceptability of the app for the first time. As the study was conducted at secondary care centers from Portugal and Spain, results may not be generalized to other settings or countries. An updated version of the app is being tested in Portuguese primary care centers. Participation in the study was restricted to patients who have access to a smartphone. Although smartphones are increasingly prevalent in patients with asthma [13], this requirement excluded otherwise eligible patients and limits the generalizability to smartphone owners. Using an app to improve treatment adherence will not be a solution for all patients, as happens with most, if not all, approaches. With this study, previous ones, and others planned it should be possible to identify those patients who may benefit from InspirerMundi app strategies. The use of two methods to calculate medication adherence may also be seen as a limitation of the study, yet it was a mitigation plan to overcome the absence of a standardized method to measure adherence [9].

### Conclusion

In conclusion, the InspirerMundi app was feasible and acceptable to monitor medication adherence in adolescents and adults with asthma. Based on patient feedback, redesign of the therapeutic plan registration and the medication detection tool have already been implemented, and other changes to the app are being considered. This study is part of a continuous development cycle that will continue with its multiple improvement and evaluation phases grounded on the interaction with end users, aiming to produce a patient-centered and engaging mHealth asthma app.

## Abbreviations

CARAT: Control of Allergic Rhinitis and Asthma Test
EQ-5D: EuroQol 5-Dimension questionnaire
GINA: Global Initiative for Asthma
mHealth: mobile health
STROBE: Strengthening the Reporting of Observational Studies in Epidemiology
VAS: visual analog scale
